# Low sodium diet for gastric cancer prevention in the United States: Results of a Markov model

**DOI:** 10.1002/cam4.3615

**Published:** 2020-12-01

**Authors:** Judith Kim, Aaron Oh, Han Truong, Monika Laszkowska, M. Constanza Camargo, Julian Abrams, Chin Hur

**Affiliations:** ^1^ Division of Digestive and Liver Diseases Department of Medicine Columbia University Irving Medical Center New York NY USA; ^2^ Division of General Medicine Department of Medicine Columbia University Irving Medical Center New York NY USA; ^3^ Department of Medicine, Gastroenterology, Hepatology, and Nutrition Service Memorial Sloan Kettering Cancer Center New York NY USA; ^4^ Division of Cancer Epidemiology and Genetics National Cancer Institute Rockville MD USA

**Keywords:** DASH diet, gastric cancer, low sodium diet, Markov model

## Abstract

**Background and Aims:**

High sodium consumption has been associated with an increased risk of gastric cancer. The mean daily sodium intake in the United States substantially exceeds the national recommended amount. The low sodium‐DASH diet has been shown to decrease the risk of cardiovascular disease in the United States, but its impact on gastric cancer has not been well studied. We therefore aimed to model the impact and cost‐effectiveness of the low sodium‐DASH diet for gastric cancer prevention in the U.S. population.

**Methods:**

A Markov cohort state‐transition model was developed to simulate the impact of the low sodium‐DASH diet on gastric cancer outcomes for the average 40‐year‐old in the United States compared to no intervention. Primary outcomes of interest were gastric cancer incidence and incremental cost‐effectiveness ratios (ICER).

**Results:**

Our model found that compared to the no intervention cohort, the risk of gastric cancer decreased by 24.8% for males and 21.2% for females on the low sodium‐DASH diet. 27 cases and 14 cases per 10,000 individuals were prevented for males and females, respectively, in the intervention group. The ICER for the low sodium‐DASH diet strategy was $287,726 for males and $423,878 for females compared to the no intervention strategy.

**Conclusions:**

Using a Markov model of gastric cancer risk, we found that adherence to a low sodium‐DASH diet could decrease the risk of gastric cancer. This intervention was not cost‐effective due to the high cost of a low sodium‐DASH accordant diet, but significantly improved for high‐risk populations and when the cost of the diet became slightly more affordable.

## Graphical abstract

Using a Markov model of gastric cancer risk, we found that adherence to a low sodium‐DASH diet decreases the risk of gastric cancer in the U.S. population. This intervention was not cost‐effective due to the high cost of a low sodium‐DASH accordant diet, but significantly improved for certain high‐risk populations and when the cost of the diet became slightly more affordable.

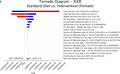

## 1 INTRODUCTION

Gastric cancer is the fifth most commonly diagnosed cancer and third leading cause of cancer death in the world. In the United States, there are an estimated 27,500 new cases annually and 11,000 deaths.^1^ *H. pylori* (Hp) is a well‐established risk factor for gastric cancer that is thought to initiate the carcinogenesis cascade. While Hp infection affects up to 50% of the world, only about 1% of the population develops gastric cancer.^2^ Other risk factors that may create a susceptible environment for carcinogenesis have not been as well characterized.

Significant geographic variations in the incidence of gastric cancer suggest that environmental factors, including diet, affect cancer risk. High salt consumption, in particular, has been associated with an increased risk of gastric cancer in prospective cohort studies.^3^, ^4^ In one study, higher median urine salt excretion level was correlated with an increased mortality rate of stomach cancer.^5^ In vitro and in vivo studies have also described mechanisms by which salt may influence gastric carcinogenesis. Cultivation of Hp in high‐salt conditions leads to alterations in the proteome, including increased expression of CagA, a known oncogenic protein.^6^, ^7^ Salt also increases mucosal inflammation and Hp colonization in murine models.^8^ In 2016, the World Cancer Research Fund concluded that greater consumption of salt preserved foods is probably a cause of gastric cancer based on the assessment of epidemiologic and biologic data.^9^

In the United States, the average daily sodium intake is 3410 mg, which substantially exceeds the daily 2300 mg recommended by national dietary guidelines.^10^ Interventions to reduce dietary sodium intake could modify the risk of gastric cancer, which continues to have a poor 5‐year survival rate of 31% in the United States.^1^ High salt consumption has also been shown to increase the risk of cardiovascular disease.^11^, ^12^ One study examining national dietary habits and disease risk found that excessive sodium intake was associated with the highest proportion of cardiometabolic deaths in the United States.^13^ The low sodium‐DASH diet has been shown to decrease daily sodium intake and to lower systolic blood pressure in the U.S. population and has been well studied.^14^, ^15^ Several models have also evaluated the cost‐effectiveness of population‐wide reductions in dietary salt on cardiovascular disease.^16^, ^17^, ^18^ The impact of dietary salt reduction has not been well‐assessed on gastric cancer outcomes, which is more difficult to assess in a clinical trial given the lower incidence of gastric cancer and longer progression time.

In this study, we aimed to model the impact and cost‐effectiveness of the low sodium‐DASH diet on gastric cancer incidence and mortality in the U.S. population.

## 2 METHODS

### 2.1 Model design

We developed a state‐transition (Markov) cohort‐level model using TreeAge Pro (TreeAge 2020) to estimate the impact of a low sodium‐DASH diet on gastric cancer outcomes. The model was calibrated such that it reproduced U.S. rates of gastric cancer. The model cohort started at age 40 and cycled annually until age 100 or death. After initial analyses, we evaluated our model inputs by performing sensitivity analyses varying the cost and utility of low sodium‐DASH diet and gastric cancer, diet adherence rates, and risk of gastric cancer. Given this was a mathematical model, institutional review board approval was determined to not be necessary.

### 2.2 Management strategies

**FIGURE 1:**
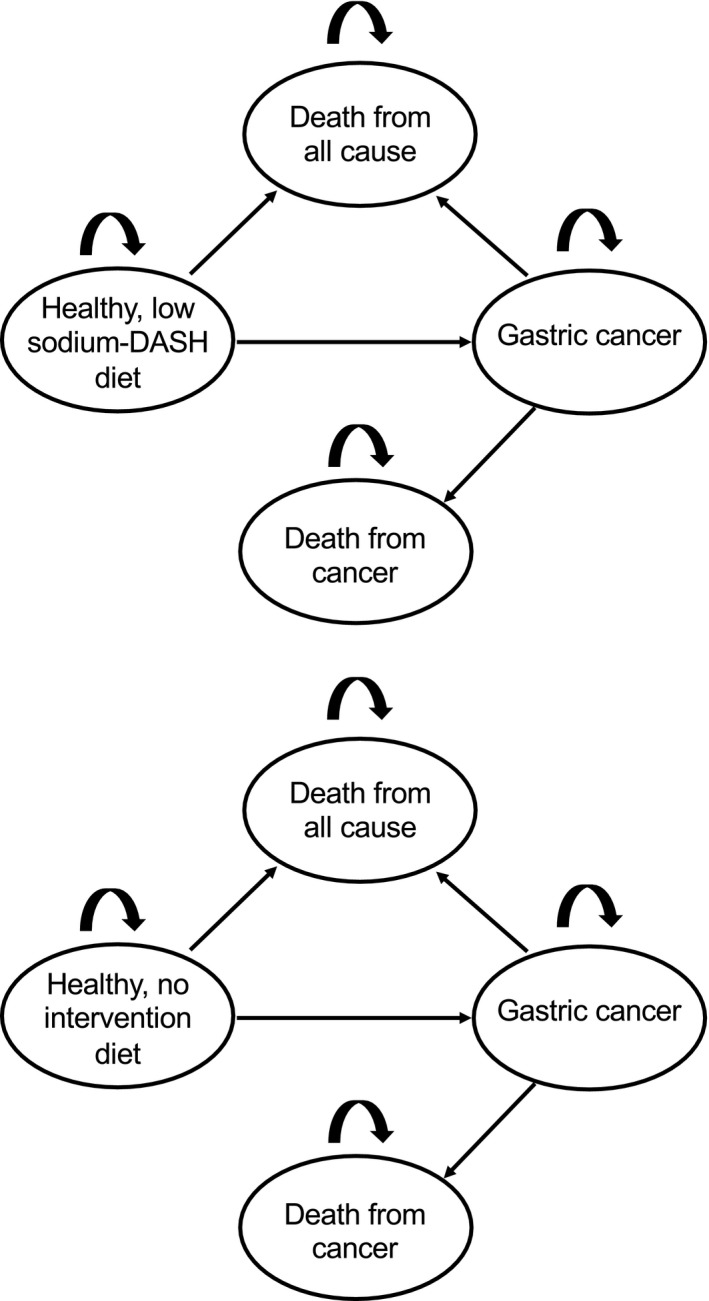
Model schematic for no intervention and dietary intervention strategies

The intervention strategy was based on the low sodium‐DASH diet used by the DASH‐sodium collaborative research group in prior cardiovascular research studies.^14^ The health states in our model included healthy, gastric cancer diagnosis, death from gastric cancer, and death due to all‐cause mortality (Figure 1).

In the natural history (no intervention) model, patients were distributed into tertiles based on the mean daily sodium intake of the U.S. population. Subjects began the model in the healthy state and could progress to gastric cancer; patients could either die from gastric cancer or all‐cause mortality. In the intervention cohort, subjects were distributed into tertiles based on expected decrease in daily sodium intake while on the low sodium‐DASH diet. For the base‐case analysis, we assumed 100% adherence and subjects remained on this diet until age 100 or death. Risks of gastric cancer and all‐cause mortality were also applied to the intervention group.

### 2.3 Outcomes

Lifetime gastric cancer incidence and incremental cost‐effectiveness ratios (ICER) were the primary outcomes of interest. Cancer mortality rate and quality‐adjusted life years (QALY) were also determined. A willingness to pay (WTP) threshold of $100,000/QALY was used to determine cost‐effectiveness.

### 2.4 Model inputs

Model parameters and ranges used in sensitivity analyses are summarized in Table 1. Annual probabilities of gastric cancer incidence and mortality were estimated based on published literature on 10‐year‐incidence and 5‐year survival rates of gastric cancer for 40‐year‐olds in the United States.^1^ All‐cause mortality rates were determined by CDC life tables. Tertiles of daily sodium intake were derived from national estimates of the dietary intake of the U.S. population.^19^ The risk of gastric cancer based on salt intake was derived from published literature (Table 1).^3^, ^9^

**TABLE 1:** Model inputs

Parameter	Base‐case estimate male	Range for sensitivity analysis	Base‐case estimate female	Range for sensitivity analysis	Distribution	Sources
Baseline age	40	—	40	—	—	Assumption
All‐cause mortality	Life tables	—	Life tables	—	—	CDC Life Tables
Lifetime GC incidence (%)	1.09	—	0.66	—	β	SEER 2014–2016^1^
Annual probability of GC	0.021	(0.016–0.026)	0.012	(0.009–0.014)	β	SEER 2014–2016^1^
GC annual mortality, Ages <45	0.194	(0.146–0.243)	0.194	(0.146–0.243)	β	SEER 2009–2015^1^
GC annual mortality, Ages 45–54	0.189	(0.141–0.234)	0.188	(0.485–0.808)	β	SEER 2009–2015^1^
GC annual mortality, Ages 55–64	0.191	(0.143–0.238)	0.191	(0.143–0.238)	β	SEER 2009–2015^1^
GC annual mortality, Ages 65–74	0.192	(0.144–0.240)	0.192	(0.143–0.240)	β	SEER 2009–2015^1^
GC annual mortality, Ages 75+	0.249	(0.187–0.311)	0.249	(0.187–0.311)	β	SEER 2009–2015^1^
Daily mean sodium intake (mg/d +/‐ SD)	4090 +/‐ 1983	—	3073 +/‐ 1360	—	—	NHANES 2016^19^
Daily mean sodium intake on DASH diet (mg/d +/‐ SD)	1500 +/‐ 1000	—	1500 +/‐ 1000	—	—	Clinical trial^14^
GC risk ratio of highest tertile of salt intake	1.68	(1.26–2.1)	1.68	(1.26–2.1)	Uniform	WCRF 2016, meta‐analysis^3^, ^9^
GC risk ratio of intermediate tertile of salt intake	1.41	(1.06–1.76)	1.41	(1.06–1.76)	Uniform	WCRF 2016, meta‐analysis^3^, ^9^
Cost GC, initial year, Ages <65 ($)	111,682.24	(55,841.12–223,364.48)	101,180.00	(50,590.00–202,360.00)	γ	National Cancer Institute^20^
Cost GC, initial year, Ages 65 ($)	93,068.13	(46,534.07–186,136.26)	84,316.86	(42,158.43–168,633.72)	γ	National Cancer Institute^20^
Cost GC, continuing care, Ages <65 ($/year)	5079.70	(2539.85–10,159.40)	4717.88	(2358.94–9435.76)	γ	National Cancer Institute^20^
Cost GC, final year, Ages <65 ($)	190,631.13	(95,315.57–381,262.26)	184,629.68	(92,314.84–369,259.36)	γ	National Cancer Institute^20^
Cost GC, final year, Ages 65 ($)	127,087.42	(63,543.71–254,174.84)	123,087.24	(61,543.62–246,174.48)	γ	National Cancer Institute^20^
Cost of low sodium‐DASH accordant diet ($/year)	3299.60	(1649.80–6599.20)	2419.95	(1209.98–4839.90)	γ	National Food Price Database^21^
Cost of intermediate accordant low sodium‐DASH diet ($/year)	3014.90	(1507.45–6029.80)	2211.90	(1105.95–4423.8)	γ	National Food Price Database^21^
Cost of least accordant low sodium‐DASH diet ($/year)	2766.70	(1383.35–5533.40)	2029.40	(1014.70–4058.80)	γ	National Food Price Database^21^
Utility healthy state	1	—	1	—	—	CEA registry^22^
Utility GC	0.68	0.58–0.78	0.68	0.58–0.78	β	CEA registry^22^
Utility low sodium‐DASH diet	0.99	0.89–1.0	0.99	0.89–1.0	β	Assumption
Adherence to diet (%)	100	10–100	100	10–100	β	Assumption

Abbreviations: GC, gastric cancer; SD, standard deviation.

### 2.5 Costs

We assessed costs from a third‐party payer perspective and discounted them at 3% per year. The model included gastric cancer treatment costs that varied with age and were divided into first year, continuing care, and final year of death. The model also included estimated costs of diets that were most accordant, intermediate accordant, and least accordant with the low sodium‐DASH diet based on prior literature. Cost estimates from prior years were converted to 2020 U.S. dollars using the Consumer Price Index (U.S. Bureau of Labor Statistics).

### 2.6 Utilities

Quality of life utility values relating to healthy and gastric cancer states were incorporated in our model. Utility decrement due a low sodium‐DASH diet was assumed to be 0.99 for the first year of diet. Quality adjusted life years were discounted at an annual rate of 3%.

### 2.7 Sensitivity analysis

One‐way deterministic sensitivity analyses were conducted by varying parameters across the ranges specified in Table 1. In addition, a probabilistic sensitivity analysis (PSA) was performed to address parameter uncertainty. PSAs were performed by varying all inputs according to the distributions in Table 1 using Monte Carlo simulations with 100,000 reiterations.

## 3 RESULTS

In the natural history (no intervention) cohort, the lifetime probability of gastric cancer for males and females at or older than 40 years of age with no intervention was 1.09% and 0.66%, respectively, which was calibrated to reflect rates of gastric cancer incidence in published literature for an average 40‐year‐old in the United States. In the model, gastric cancer was the cause of mortality in 0.97% of males and 0.59% of females of the overall population. QALYs associated with the base‐case were 22.1 for males and 23.4 for females. Unadjusted life‐years or survival with no intervention were 38.5 for males and 42.4 for females.

In the intervention cohort, the lifetime probability of gastric cancer for males and females on the intervention diet were 0.82% and 0.52%, respectively, which was decreased compared to the natural history cohort (Table 2). Gastric cancer was the cause of mortality in 0.73% for males and 0.46% for females. QALYs associated with base case were 22.1 for males and 23.4 for females. Unadjusted life‐years with intervention were 38.5 for males and 42.5 for females.

**TABLE 2:** Base‐case model outputs

	Base‐case males	Base‐case females
Initial lifetime probability of GC (%)	1.09	0.66
Post‐intervention probability of GC (%)	0.82	0.52
Post‐intervention risk reduction (%)	24.8	21.2
Cases prevented per 10,000 individuals	27	14
Initial lifetime GC mortality rate (%)	0.97	0.59
Post‐intervention GC mortality (%)	0.73	0.46
Deaths prevented per 10,000 individuals	24	13
ICER ($/QALY)	287,726	423,878

Abbreviations: GC, gastric cancer; ICER, incremental cost‐effectiveness ratio; QALY, quality‐adjusted life year.

Compared to the natural history cohort, the risk of gastric cancer decreased by 24.8% for males and by 21.2% for females in the intervention cohort (Table 2). About 27 gastric cancer cases and 14 cases per 10,000 individuals were prevented for males and females, respectively, on the low sodium‐DASH diet. In addition, 24 deaths for males and 13 deaths for females from gastric cancer were prevented per 10,000 individuals. When compared to the no intervention strategy, the ICER for the low sodium‐DASH diet strategy was $287,726 for males and $423,878 for females.

**FIGURE 2:**
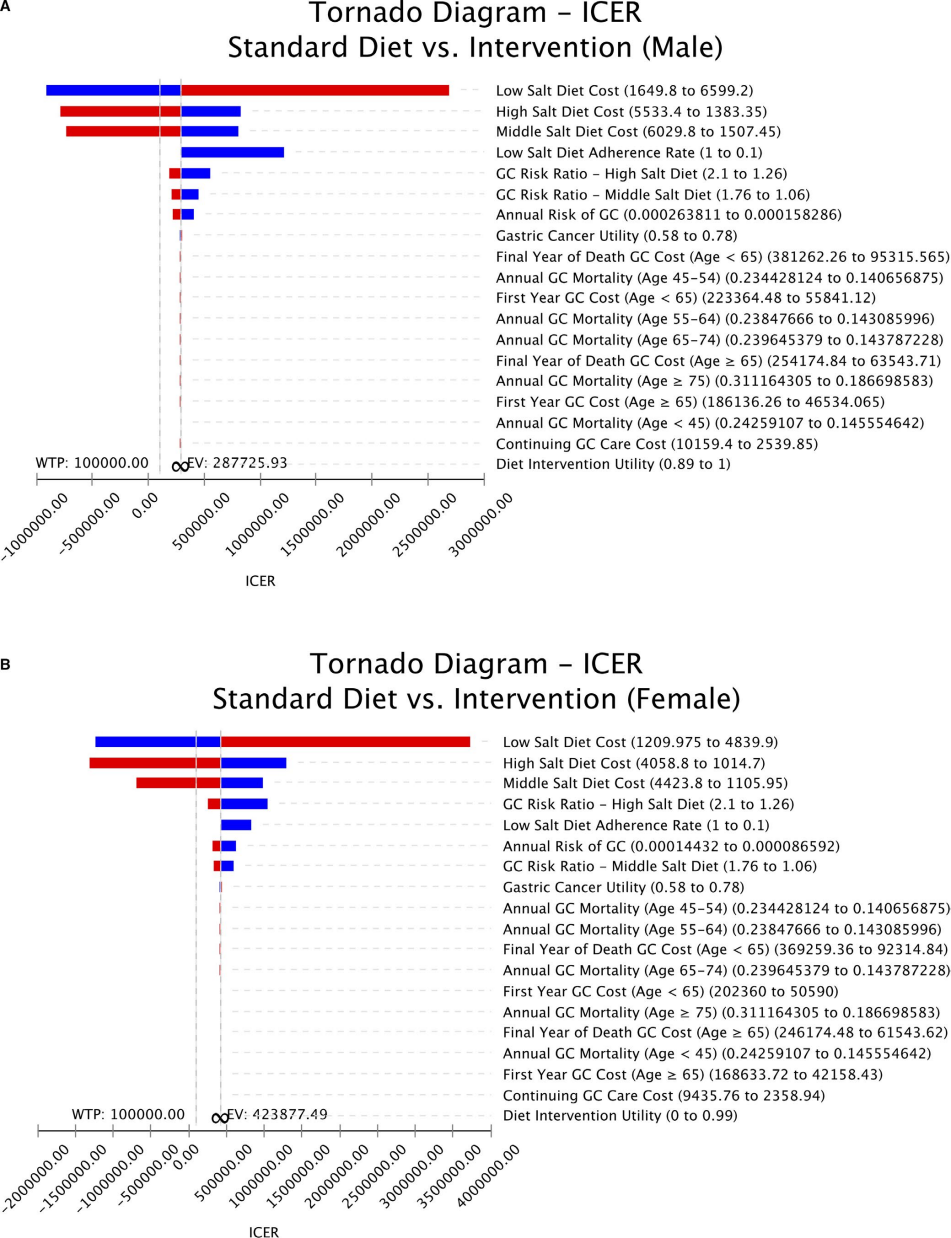
A, Tornado diagram of one‐way sensitivity analysis results for males. B, Tornado diagram of one‐way sensitivity analysis results for females

**FIGURE 3:**
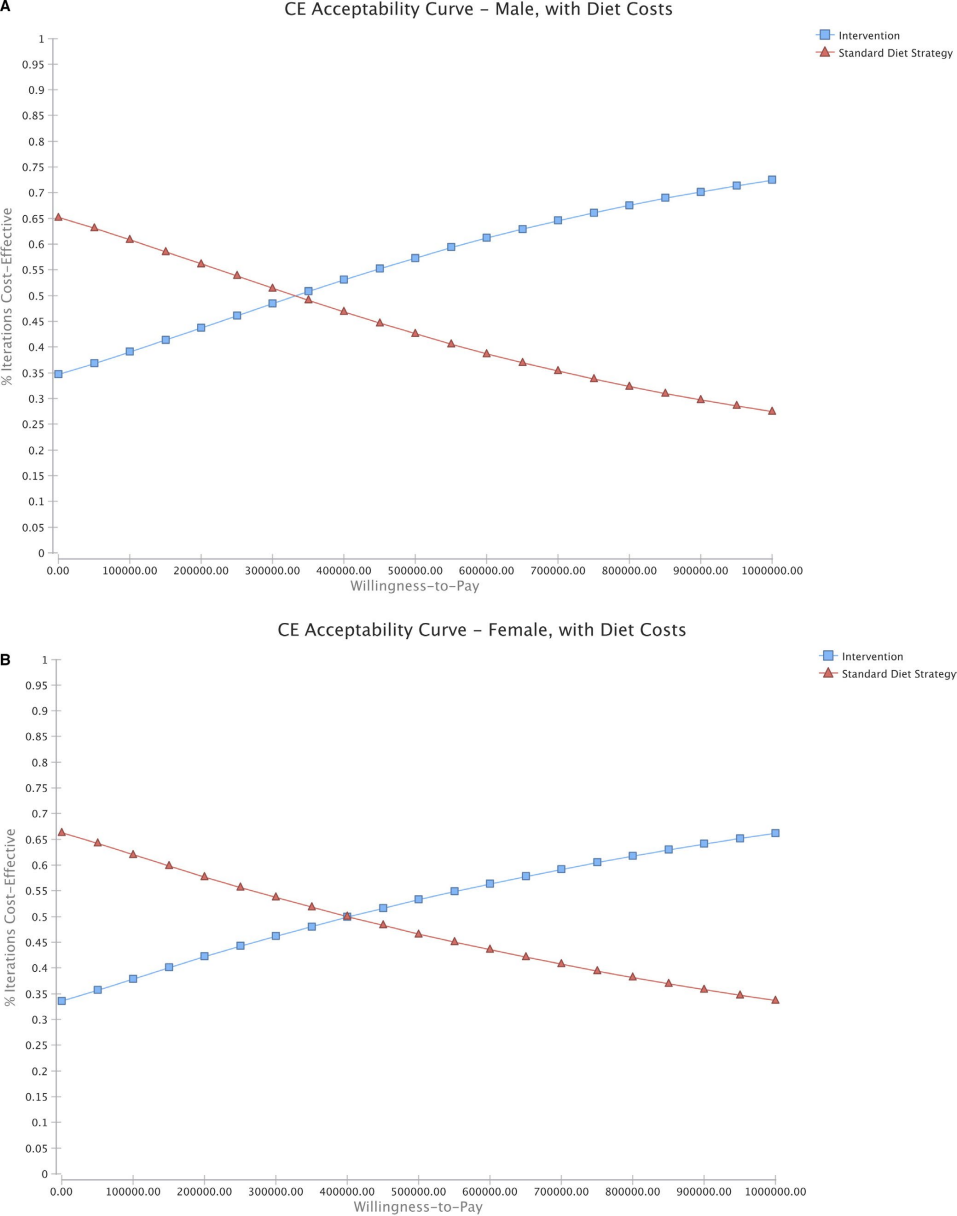
A, Cost‐effectiveness acceptability curves for males. B, Cost‐effectiveness acceptability curves for females

One‐way deterministic sensitivity analyses are presented in Figure 2. Results were most sensitive to the cost of diet for both males and females. As the annual cost of the low sodium‐DASH accordant diet decreased, the ICER approached the WTP threshold. Cost‐effectiveness acceptability curves were used to present the results of the PSA and to determine the probability of any strategy being cost effective at a given WTP (Figure 3). For males, the intervention was cost‐effective at a WTP threshold of $100,000/QALY in 39.1% of the iterations, while for females, it was cost‐effective in 37.9% of the iterations. When the annual cost of the low sodium‐DASH accordant diet decreased from $3299.60 to $3041.94 for males or from $2419.95 to $2182.73 for females, the strategy became cost‐effective at the WTP threshold. The intervention also became cost‐effective for populations with a higher probability of gastric cancer than the base‐case, at an annual probability of gastric cancer of 0.051% for males or 0.037% for females.

## 4 DISCUSSION

An unhealthy diet is an important cause of mortality in the United States. The low sodium‐DASH diet has been shown to decrease daily sodium intake and be cost‐effective through its impact on cardiovascular outcomes.^16^, ^17^, ^18^ The impact of dietary salt reduction has not been well‐assessed on gastric cancer outcomes in the U.S. population. Gastric cancer outcomes remain poor in the United States, as it is often diagnosed in advanced stages and there is no curative therapy for unresectable disease.^1^ Assessing the impact of modifiable risk factors, such as diet, on gastric cancer is important for developing preventative strategies.

Based on the results of this study, we found that adherence to a low sodium diet resulted in a lower incidence of gastric cancer cases in both males and females. There was a 24.8% decrease in gastric cancer risk for males and a 21.2% decrease for females in the intervention cohort compared to the natural history cohort. There was a 24.7% reduction in lifetime risk of dying from gastric cancer for males and 22.0% reduction for females.

Given the higher cost of a low sodium diet over a lifetime, this strategy exceeded a willingness to pay threshold of $100,000/QALY. However, there were several variables that modified the cost‐effectiveness of the intervention strategy on sensitivity analyses. The low salt‐DASH was cost‐effective for high‐risk populations who have an increased lifetime gastric cancer risk compared to the average 40‐year‐old in the United States. Based on our sensitivity analysis, the low sodium‐DASH diet would be cost‐effective for those with a lifetime risk of gastric cancer of 2.6% or higher for males or 2.1% or higher for females, which is approximately two or three times the baseline gastric cancer risk in the United States. The lifetime risk of being diagnosed with cancer for Asian/Pacific Islanders in the United States is reported to be 2.03% for males and 1.33% for females.^1^ In addition, mean sodium intake in East Asian countries, such as Japan and South Korea, is estimated to be 5000 mg/day, much higher than 3440 mg/day in the United States. Asian‐Americans also have a higher mean sodium consumption compared to the average U.S. daily intake (3850 mg vs. 3410 mg).^19^ Asian‐Americans also have the lowest proportion of individuals adherent with the recommended amount of daily sodium (2300 mg) compared to other racial groups in the United States.^23^ For Asian‐Americans or Asian immigrants in the United States, in particular, the low sodium‐DASH diet may be cost‐effective given their increased risk of gastric cancer and higher mean sodium intake.

Our sensitivity analysis also showed that when the cost of the intervention diet approached the costs of the less adherent diet, the intervention strategy was cost‐effective in reducing gastric cancer incidence. The cost of a low sodium‐DASH accordant would only need to be $21.50/month lower in order to meet the willingness to pay threshold of $100,000/QALY. The DASH accordant diet may be more expensive for several reasons. U.S. agricultural policies have historically focused on facilitating the production and marketing of highly processed food products and so less healthful foods have become cheaper and more widely available.^24^ In addition, the supply of fruits and vegetables in the United States is lower than the amount necessary to fulfill the daily servings recommended by the DASH diet, which may raise prices.^25^, ^26^ Public health and population‐based interventions to lower costs of optimal diets would help to decrease overall healthcare costs. Encouraging industry participation or taxation on high‐sodium foods are possible methods to improve access to low sodium diets. The National Salt Reduction Initiative, led by the New York City Department of Health and Mental Hygiene, was one of the first efforts in the United States in 2009 to lower sodium intake through collaboration with food manufacturers and retailers.^27^ About a quarter of the food categories met the 3‐year reduction targets through this voluntary program, and the initiative showed that sodium reductions in the regular food supply were feasible and could help to improve general public access to low sodium dietary content.

Individuals can lower their dietary sodium intake through various methods. It is important to minimize using salt while cooking or adding salt to prepared food. Processed foods should be substituted with whole foods when possible. Since more than 75% of dietary sodium comes from packaged and restaurant food, it is necessary to intentionally identify low‐sodium or sodium‐free options at grocery stores and restaurants.^27^ Processed meats and bread and bakery products have been reported to be the two highest contributors to sodium intake,^28^, ^29^ and so particular attention should be paid to these food groups.

Our analysis did not incorporate cost‐savings associated with hypertension, myocardial infarctions, strokes, or kidney disease, which are much more prevalent in the U.S. population than gastric cancer, but was beyond the scope of this analysis.^11^, ^12^, ^30^ This would make low sodium dietary interventions even more cost‐effective as disease burden decreases and associated healthcare costs are prevented. Furthermore, most prior models evaluating the impact of a low sodium diet on cardiovascular outcomes did not incorporate costs of specific diets or adherence rates as in our model.^16^, ^17^

There are several limitations to our study. Model data sources came from multiple sources and not from single cohorts. While we used data specific to the U.S. population for estimating gastric cancer incidence and mortality, diet costs, and cancer costs, we used pooled data to determine the risk of gastric cancer based on salt intake. We also assumed 100% adherence to diet in our base‐case analysis and so patients remained within their range of dietary salt intake for their lifetime. In an additional analysis, we decreased the adherence to the low salt diet from 100% to between 30% and 90%. There was an 8.3% to 22.8% reduction in risk of gastric cancer for males and 7.7% to 19.4% for females, which is lower than our base case risk reduction of 24.8% and 21.2% for males and females respectively. Adherence to a low sodium diet impacts the magnitude of gastric cancer risk reduction.

In conclusion, using a Markov model of gastric cancer incidence, we found that adherence to a low salt‐DASH diet substantially reduces the risk of gastric cancer. While this was not cost‐effective due to the higher cost of a low sodium‐DASH diet, the cost‐effectiveness improved for high‐risk populations and when costs of the diet became slightly more affordable. Clinical studies to affirm some of our model assumptions are needed, and public health measures to reduce the cost of healthy foods lower in sodium content are warranted.

## CONFLICT OF INTEREST

All authors have no relevant conflicts of interest to report.

## AUTHOR CONTRIBUTIONS

Study conceptualization (JK, AO, HT, and CH), data curation (JK, AO, and HT), formal analysis (JK and AO), investigation (JK and AO), methodology (JK and AO), writing‐original draft (JK), writing‐review and editing (JK, AO, HT, ML, MCC, JA, and CH).

## Data Availability

The data that support the findings of this study are available from the corresponding author upon reasonable request.

## References

[1] Howlader NNA , Krapcho M , Miller D , et al. SEER Cancer Statistics Review, 1975–2016. Bethesda, MD: National Cancer Institute Available from https://seer.cancer.gov/csr/1975_2016/

[2] Correa P , Piazuelo MB . Helicobacter pylori Infection and Gastric Adenocarcinoma. US Gastroenterol Hepatol Rev. 2011;7(1):59‐64. 21857882 PMC3158605

[3] D'Elia L , Rossi G , Ippolito R , Cappuccio FP , Strazzullo P . Habitual salt intake and risk of gastric cancer: a meta‐analysis of prospective studies. Clin Nutr. 2012;31(4):489‐498. 22296873 10.1016/j.clnu.2012.01.003

[4] Shikata K , Kiyohara Y , Kubo M , et al. A prospective study of dietary salt intake and gastric cancer incidence in a defined Japanese population: the Hisayama study. Int J Cancer. 2006;119(1):196‐201. 16450397 10.1002/ijc.21822

[5] Tsugane S , Tsuda M , Gey F , Watanabe S . Cross‐sectional study with multiple measurements of biological markers for assessing stomach cancer risks at the population level. Environ Health Perspect. 1992;98:207‐210. 1486851 10.1289/ehp.9298207 PMC1519596

[6] Loh JT , Friedman DB , Piazuelo MB , et al. Analysis of *Helicobacter pylori* cagA promoter elements required for salt‐induced upregulation of CagA expression. Infect Immun. 2012;80(9):3094‐3106. 22710874 10.1128/IAI.00232-12 PMC3418733

[7] Voss BJ , Loh JT , Hill S , Rose KL , McDonald WH , Cover TL . Alteration of the Helicobacter pylori membrane proteome in response to changes in environmental salt concentration. Proteomics Clin Appl. 2015;9(11–12):1021‐1034. 26109032 10.1002/prca.201400176 PMC4690801

[8] Gaddy JA , Radin JN , Loh JT , et al. High dietary salt intake exacerbates Helicobacter pylori‐induced gastric carcinogenesis. Infect Immun. 2013;81(6):2258‐2267. 23569116 10.1128/IAI.01271-12 PMC3676043

[9] World Cancer Research Fund International/American Institute for Cancer Research . Continuous Update Project Report: Diet, Nutrition, Physical Activity and Stomach Cancer.

[10] U.S. Department of Health and Human Services and U.S. Department of Agriculture . 2015–2020 Dietary Guidelines for Americans. 8th ed December 2015 Available from http://health.gov/dietaryguidelines/2015/guidelines/

[11] He FJ , Li J , Macgregor GA . Effect of longer‐term modest salt reduction on blood pressure. Cochrane Database Syst Rev. 2013 ; 10.1002/14651858.CD004937.pub2 PMC11537250 23633321

[12] Malta D , Petersen KS , Johnson C , et al. High sodium intake increases blood pressure and risk of kidney disease. From the science of salt: a regularly updated systematic review of salt and health outcomes. J Clin Hypertens. 2018;20(12):1654‐1665. 10.1111/jch.13408 PMC8030856 30402970

[13] Micha R , Penalvo JL , Cudhea F , Imamura F , Rehm CD , Mozaffarian D . association between dietary factors and mortality from heart disease, stroke, and type 2 diabetes in the United States. JAMA. 2017;317(9):912‐924. 28267855 10.1001/jama.2017.0947 PMC5852674

[14] Sacks FM , Svetkey LP , Vollmer WM , et al. Effects on blood pressure of reduced dietary sodium and the dietary approaches to stop hypertension (DASH) diet. DASH‐sodium collaborative research group. N Engl J Med. 2001;344(1):3‐10. 11136953 10.1056/NEJM200101043440101

[15] Juraschek SP , Miller ER 3rd , Weaver CM , Appel LJ . Effects of sodium reduction and the DASH diet in relation to baseline blood pressure. J Am Coll Cardiol. 2017;70(23):2841‐2848. 29141784 10.1016/j.jacc.2017.10.011 PMC5742671

[16] Smith‐Spangler CM , Juusola JL , Enns EA , Owens DK , Garber AM . Population strategies to decrease sodium intake and the burden of cardiovascular disease: a cost‐effectiveness analysis. Ann Intern Med. 2010;152(8):pp. 481–487, W170–483. 10.7326/0003-4819-152-8-201004200-00212 20194225

[17] Bibbins‐Domingo K , Chertow GM , Coxson PG , et al. Projected effect of dietary salt reductions on future cardiovascular disease. N Engl J Med. 2010;362(7):590‐599. 20089957 10.1056/NEJMoa0907355 PMC3066566

[18] Pearson‐Stuttard J , Kypridemos C , Collins B , et al. Estimating the health and economic effects of the proposed US Food and Drug Administration voluntary sodium reformulation: Microsimulation cost‐effectiveness analysis. PLoS Med. 2018;15(4):e1002551. 29634725 10.1371/journal.pmed.1002551 PMC5892867

[19] United States Department of Agriculture . What We Eat in America, NHANES 2015–2016. Available from https://www.ars.usda.gov/

[20] Mariotto AB , Yabroff KR , Shao Y , Feuer EJ , Brown ML . Projections of the cost of cancer care in the United States: 2010–2020. J Natl Cancer Inst. 2011;103(2):117‐128. 21228314 10.1093/jnci/djq495 PMC3107566

[21] Monsivais P , Rehm CD , Drewnowski A . The DASH diet and diet costs among ethnic and racial groups in the United States. JAMA Intern Med. 2013;173(20):1922‐1924. 23999924 10.1001/jamainternmed.2013.9479 PMC3856355

[22] Center for the Evaluation of Value and Risk in Health . The Cost‐Effectiveness Analysis Registry [Internet]. In: (Boston), Institute for Clinical Research and Health Policy Studies, Tufts Medical Center. Available from www.cearegistry.org

[23] Firestone MJ , Beasley JM , Kwon SC , Ahn J , Trinh‐Shevrin C , Yi SS . Asian American dietary sources of sodium and salt behaviors compared with other racial/ethnic groups, NHANES, 2011–2012. Ethn Dis. 2017;27(3):241‐248. 28811735 10.18865/ed.27.3.241 PMC5517142

[24] Popkin BM . Agricultural policies, food and public health. EMBO Rep. 2011;12(1):11‐18. 21151043 10.1038/embor.2010.200 PMC3024136

[25] Bertoni AG , Whitt‐Glover MC . DASH for less cash? JAMA Intern Med. 2013;173(20):1924‐1925. 23999885 10.1001/jamainternmed.2013.9163 PMC4717901

[26] Siegel KR , Ali MK , Srinivasiah A , Nugent RA , Narayan KM . Do we produce enough fruits and vegetables to meet global health need? PLoS One. 2014;9(8):e104059. 25099121 10.1371/journal.pone.0104059 PMC4123909

[27] Curtis CJ , Clapp J , Niederman SA , Ng SW , Angell SY . US food industry progress during the national salt reduction initiative: 2009–2014. Am J Public Health. 2016;106(10):1815‐1819. 27552265 10.2105/AJPH.2016.303397 PMC5024394

[28] Centers for Disease C, Prevention . Vital signs: food categories contributing the most to sodium consumption – United States, 2007–2008. *MMWR Morb Mortal Wkly Rep* 2012;61(5):92‐98. 22318472

[29] Ni Mhurchu C , Capelin C , Dunford EK , Webster JL , Neal BC , Jebb SA . Sodium content of processed foods in the United Kingdom: analysis of 44,000 foods purchased by 21,000 households. Am J Clin Nutr. 2011;93(3):594‐600. 21191142 10.3945/ajcn.110.004481 PMC3561609

[30] Smyth A , Griffin M , Yusuf S , et al. Diet and major renal outcomes: a prospective cohort study. The NIH‐AARP diet and health study. J Ren Nutr. 2016;26(5):288‐298. 26975776 10.1053/j.jrn.2016.01.016

